# Chronic diseases and depressive symptoms in China: a psychosocial mechanism perspective

**DOI:** 10.3389/fpubh.2025.1696199

**Published:** 2025-10-27

**Authors:** Qingqing Xu, Ruizhe Shang, Xia Jiang

**Affiliations:** School of Economics, Qingdao University, Qingdao, China

**Keywords:** chronic diseases, depressed symptoms, income satisfaction, job safety satisfaction, social cognitive capital, ordinal logistic model

## Abstract

**Introduction:**

Although the co-morbidity of chronic diseases and depression has been widely documented, its psychosocial mechanisms have not been systematically explored. This study analyzed the impact of chronic disease on depression, while emphasizing the mediating roles that income satisfaction, job safety satisfaction, and social cognitive capital play in the psychosocial mechanism.

**Methods:**

Using the 2022 China Family Tracking Survey (CFPS) data, this study conducted a baseline regression using an ordered logit model, and explored the pathways of chronic illnesses on depressive symptoms through mechanism tests and heterogeneity analyses (*n* = 7,896).

**Results:**

Chronic conditions are associated with a considerable increase in the risk of depressive symptoms (OR = 1.671, *p* < 0.01). Mechanistic analyses showed that income satisfaction, job safety satisfaction and social cognitive capital all played partial mediating roles. Heterogeneity analyses indicated that the impact of chronic illness on depression was stronger among males, individuals with less robust social support, and non-users of the Internet.

**Discussion:**

This research reveals that chronic illness constitutes a notable risk factor for depression and underscores its indirect impacts on mental health through three psychosocial pathways: financial strain, job safety, and insufficient interpersonal trust. The results emphasize the importance of integrating socioeconomic support, workplace improvement and digital health resources in chronic disease management to mitigate the risk of depression.

## 1 Introduction

As a major global public health issue, chronic diseases not only inflict long-term harm on patients’ physical health but also exert a profound influence on their psychological well-being. Studies have revealed a strong connection between chronic pain and depression. In addition, patients with chronic diseases—including diabetes, cancer, stroke, chronic liver disease, kidney disease, and COPD—have a notably higher incidence of mental disorders and a greater likelihood of developing depression (1–12). Chronic illnesses not only lead to a decrease in the patient’s ability to perform daily living tasks, but may also exacerbate the depressive condition (13). The long duration of the disease, complex treatment, and numerous complications not only bring financial burden to the family, but also may induce or aggravate depressive symptoms through physiological mechanisms such as neuroendocrine dysregulation (14). Conversely, depression can itself contribute to a higher susceptibility to chronic illnesses, adversely affecting their prognosis and diminishing the overall quality of life. This bidirectional interaction—where chronic diseases exacerbate depression and depression increases susceptibility to chronic diseases—often creates a self-perpetuating cycle that is challenging to overcome (15, 16). Beck’s cognitive model of depression posits that patients with chronic diseases are forced to abandon valued activities due to illness-related limitations. This creates a profound sense of psychological loss and burden, representing a key mechanism in the onset of depression (17). Consequently, it is essential—from both clinical and public health angles—to detect and manage depression early in patients suffering from chronic diseases.

More notably, compared to a single chronic disease, co-morbidity typically implies a longer disease course, more complex treatment processes, and poorer health outcomes. Regarding chronic comorbidities and depression, a meta-analysis indicates that those with two or more chronic conditions experience an approximately twofold higher risk of depression than individuals without any (6). The coexistence of multiple diseases not only leads to diminished physical function and deteriorated quality of life but also significantly increases mortality risk (18, 19). The impact of chronic diseases extends beyond the medical sphere, profoundly affecting psychological and social functioning. Patients and their families often bear heavy economic burdens and psychological stress (20). Studies reveal that chronic illnesses have become the primary driver behind the reduction in health status among people in China (21). Depression, as a highly disabling mental disorder, can cause emotional disturbances, sleep problems, and even suicidal behavior, further exacerbating the disease burden. By 2017, depression had affected over 258 million people globally, including 56 million in China (22, 23).

Although the comorbidity of chronic diseases and depression has been widely documented (9, 24–26), its underlying mechanisms—particularly the psychosocial pathways—remain insufficiently explored. Existing research predominantly focuses on biological mechanisms such as inflammatory responses and neuroendocrine dysregulation (27, 28), while rarely conducting systematic analyses of the mediating processes through which chronic diseases lead to depression from multidimensional perspectives including social, psychological, and behavioral factors. For instance, patients with limited physical activity often become more dependent on others, experience reduced social engagement, and exhibit diminished interpersonal trust (29). These shifts in psychosocial factors may serve as crucial mediators for depression onset. Conversely, modern information technologies like the internet provide chronic disease patients with novel avenues for social support and information access (30). Research indicates that internet users exhibit lower chronic disease risk (31), as patients can utilize online platforms for social interaction, medical information retrieval, and recreational activities—thereby enhancing psychological well-being. Therefore, the psychosocial mechanisms within the digital society context warrant further exploration. Economic pressures also represent a significant mediating factor. Most chronic diseases require long-term management or lifelong treatment, imposing sustained financial burdens and labor force losses (32, 33). Low income coupled with high expenditures may lead to a loss of perceived control over life, thereby inducing feelings of helplessness and depressive symptoms. Consequently, constructing a comprehensive psychosocial mechanism analysis framework encompassing socioeconomic status, job safety, and social cognitive capital not only helps elucidate the complex pathways linking chronic diseases and depression but also provides a theoretical foundation for developing targeted psychological interventions.

This study employs an ordered logit model to investigate the impact of chronic diseases on depressive symptoms, placing emphasis on the mediating effects of income satisfaction, job safety, and interpersonal trust. Furthermore, the study performed heterogeneity analyses across three dimensions—gender, social support, and internet usage—to explore variations in the chronic illness-depression association among different subgroups. The results provide a scientific foundation for developing psychological interventions in chronically ill patients, emphasizing the integration of psychosocial support into long-term care to enhance well-being and alleviate the combined burden of physical and mental health conditions.

## 2 Datasets

### 2.1 Sample selection

This study makes use of data from the 2022 China Family Panel Studies (CFPS), which is a nationally representative survey carried out by the Institute of Social Science Survey affiliated with Peking University (34). Fieldwork was conducted between May and December 2022, which encompasses four key survey instruments (community, household, adult, and child modules) within the framework of stratified multi-stage probability sampling.

**Figure 1:**
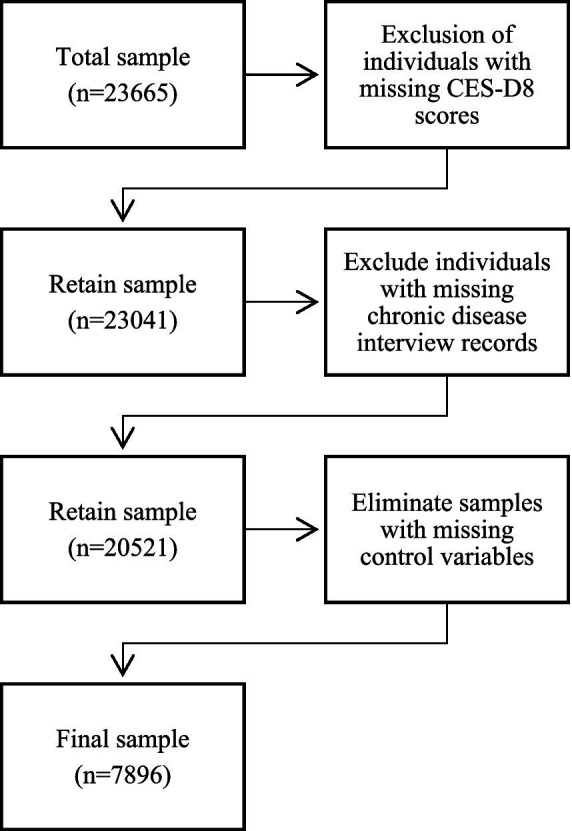
Flowchart of sample screening.

During the sample selection process, the study included individuals of legal working age^1^ (males: aged 18–60; females: aged 18–55) and excluded individuals with unclear educational characteristics and mental health data. The analytical framework incorporated 7,896 eligible participants meeting inclusion criteria, with sample attrition pathways and exclusion rationales systematically visualized in Figure 1 to ensure methodological transparency.

### 2.2 Measure

#### 2.2.1 Explained variable: depressive symptoms

Depressive symptoms were evaluated using the 8-item Center for Epidemiologic Studies Depression Scale, a shortened version of the original CES-D created by Radloff (35). The scale measures the frequency of eight symptoms experienced in the past week, including: low mood, trouble starting tasks, sleep disturbances, lack of positive feelings, social isolation, reduced happiness, prolonged sadness, and loss of motivation. Responses were scored on a 4-point scale, ranging from 1 (rarely; <1 day) to 4 (most of the time; 5–7 days). Two positive items (e.g., happiness) were reverse-scored. The total score ranges from 8 to 32, with higher scores indicating more severe depressive symptoms.

#### 2.2.2 Explanatory variable: chronic

Chronic disease prevalence was measured in this study using the following question: “Have you received a diagnosis of a chronic disease from a doctor in the past 6 months?” This variable is binary, coded as 1 for “yes” and 0 for “no.” This self-reported method based on physician diagnosis accurately identifies clinically confirmed chronic disease status within the past 6 months, providing a reliable empirical foundation for subsequent analysis.

#### 2.2.3 Mechanism variables

To further elucidate how chronic diseases influence the onset and progression of depression through psychosocial mechanisms, this study introduces income satisfaction, job satisfaction, and social cognitive capital as mediating variables, constructing a multidimensional mechanism analysis framework. These variables aim to explain the psychological adaptation challenges chronic disease patients may face when confronting long-term health pressures—and their ultimate impact on depressive states—not only from the perspective of objective material conditions but also from subjective cognitive and emotional experiences.

First, income satisfaction serves as a key indicator reflecting individuals’ subjective evaluation of their economic status. Its level directly relates to patients’ psychological resilience when coping with additional medical expenses, income reduction, or diminished work capacity stemming from chronic illness. This study employs a 5-point scale to measure this indicator, with scores ranging from 1 (very dissatisfied) to 5 (very satisfied). Chronic illness may reduce labor participation rates and work efficiency, thereby impacting personal and household finances. Low income satisfaction may intensify patients’ perceived economic strain, inducing feelings of helplessness and anxiety, ultimately increasing depression risk. Thus, income satisfaction plays a crucial mediating role between chronic illness and depression: chronic illness constrains economic capacity, lowers income satisfaction, and subsequently affects mental health status.

Second, job safety satisfaction serves as another critical psychosocial mediating variable. This study measured it using the CFPS questionnaire item “Are you satisfied with the safety of your current job?.” This variable reflects individuals’ subjective perceptions regarding workplace physical safety conditions, protection against physical hazards, and labor safety measures. Chronic disease patients often exhibit heightened sensitivity to safety risks in the workplace due to compromised health. For instance, physical discomfort may increase the risk of operational errors or accidents, or physical limitations may hinder their ability to handle high-risk tasks. Such concerns and dissatisfaction regarding safety issues significantly increase psychological burden, triggering anxiety and tension, which in turn indirectly exacerbate depressive symptoms. Therefore, perceived workplace safety acts as a conduit between chronic illness and depressive symptoms.

Third, social cognitive capital—reflecting core trust and social cognition—is defined as the “tendency to trust others.” It adopts a binary coding method: “trust” is coded as 1, and “distrust” as 0. Chronic disease patients, due to prolonged illness, may experience reduced social interaction and even impaired interpersonal trust stemming from disease stigma. Trust constitutes a core component of social capital. Higher social cognitive capital facilitates effective acquisition of social support, helping patients better integrate into social networks and strengthen psychological resilience. Conversely, patients who tend to distrust others are more likely to engage in social avoidance and experience emotional isolation, thereby amplifying the negative psychological impacts of chronic illness. Thus, social cognitive capital acts as a cognitive mediator between chronic illness and depression. A lack of trust may intensify patients’ feelings of helplessness and loneliness, subsequently increasing the likelihood of depression.

#### 2.2.4 Control variables

Given the numerous factors influencing individual depressive symptoms, the following four control variables were selected based on relevant studies (36, 37). (1) Individual characteristics: age, gender, marital status, and education. (2) Employment characteristics: labor contract status and weekly working hours. (3) Health behaviors: smoking in the past month and frequent drinking (≥3 times/week). (4) Socioeconomic status: self-rated local income level. Variable descriptions are provided in Table 1.

**Table 1:** Measurement of variables.

Variable	Abbreviation	Measurement	Source
Depressive symptoms	dp	Depressive symptoms was quantified using a cumulative metric (potential range: 8–32 aggregate points), with elevated scores being positively associated with clinical depression severity	CFPS Individual Questionnaire QN406, QN407, QN411, QN412, QN 414, QN416, QN418, QN420
Chronic disease	chr	0 = Without chronic disease, 1 = With chronic disease	CFPS Individual Questionnaire QP401
Age	age	Actual age of respondents	CFPS Individual Questionnaire A001
Gender	gender	0 = Female, 1 = Male	CFPS Individual Questionnaire QA002
Marital status	marry	1 = Unmarried, 2 = Married, 3 = Cohabiting, 4 = Divorced, 5 = Widowed	CFPS Individual Questionnaire QEA0
Education	edu	No formal education = 0, Primary school = 6, Junior high school = 9, High school/technical school = 12, College = 14, Bachelor’s degree = 16, Master’s degree = 19, Doctoral degree = 23	CFPS Individual Questionnaire W01
Working hours	hour	Actual weekly working hours	CFPS Individual Questionnaire QG6
Labor contract	lc	0 = Not signed, 1 = Signed	CFPS Individual Questionnaire QG5
Smoking	smok	0 = Did not smoke, 1 = Smoked	CFPS Individual Questionnaire QQ201
Alcohol consumption	drink	0 = Drank alcohol ≤3 times per week, 1 = Drank alcohol >3 times per week	CFPS Individual Questionnaire QQ301
Local income level	sincome	Scores ranging from 1 (low) to 5 (high)	CFPS Individual Questionnaire QN8011
Income satisfaction	inc	Scores ranging from 1 (low) to 5 (high)	CFPS Individual Questionnaire QG401
Job safety Satisfaction	saf	Scores ranging from 1 (low) to 5 (high)	CFPS Individual Questionnaire QG405
Social cognitive capital	ren	0 = Be cautious when interacting with people, 1 = Most people can be trusted	CFPS Individual Questionnaire QN1001

## 3 Methods

### 3.1 Benchmark regression model

In this study, data were cleaned and analysed using Stata 17.0 software. Since the explanatory variable (dp) is an 8–12 ordered variable, this paper uses an ordered logit model rather than OLS (Ordinary Least Squares) to evaluate the link between the explained variable (dp) and the explanatory variable (chr) (37–39). Benchmark regression model is shown below:


$$dp_{\mathrm{ijm}}=\alpha +\mathrm{\beta ch}r_{\mathrm{ijm}}+\sum_{k=1}^{10}\theta_{k}Z_{k}+\gamma_{m}+\varepsilon_{\mathrm{ijm}}$$

Where i denotes an individual, j denotes a province, and m denotes an occupation; dp is an explanatory variable representing an individual’s level of depression; chr is a dummy variable for chronic; $Z_{k}$ represents control variables including individual characteristics, job characteristics, health behavior, biomedical status, and socioeconomic prositioning; $\gamma_{m}$ is a dummy variable for an occupation; and $\varepsilon_{\mathrm{ijm}}$ is a random error term.

### 3.2 Mechanism model

To examine how chronic diseases influence depressive symptoms through psychosocial factors, we employed the following mechanistic model for regression analysis.


(1) $$M_{\mathrm{ijm}}=\alpha_{1}+\beta_{1}\mathrm{ch}r_{\mathrm{ijm}}+\sum_{k=1}^{10}\theta_{k}Z_{k}+\gamma_{m}+\varepsilon_{\mathrm{ijm}}$$

(2) $$dp_{\mathrm{ijm}}=\alpha_{2}+\beta_{2}\mathrm{ch}r_{\mathrm{ijm}}+\sum_{k=1}^{10}\theta_{k}Z_{k}+\gamma_{m}+\varepsilon_{\mathrm{ijm}}$$

(3) $$\begin{array}{c} dp_{\mathrm{ijm}}=\alpha_{3}+\beta_{3}\mathrm{ch}r_{\mathrm{ijm}}+\beta_{4}M_{\mathrm{ijm}}+\sum_{k=1}^{10}\theta_{k}Z_{k}+\gamma_{m}+\varepsilon_{\mathrm{ijm}} \end{array}$$

Equation 1 first examines whether chronic has an effect on mechanism variables, Equation 2 further examines the role of mechanism variables on depressive symptoms, and finally Equation 3 is designed to examine whether the inclusion of mechanism variables attenuates the effect of chronic diseases on depressive symptoms. Where $M_{\mathrm{ijm}}$ represents mechanism variables including income satisfaction, job safety satisfaction, and social cognitive capital.

## 4 Results

### 4.1 Descriptive statistics

The average score for depressive symptoms was 13.65 (SD = 3.88). This mean indicates that the overall depression level of the sample population falls below the theoretical median (20 points), presenting an overall characteristic of mild depressive tendencies. The core independent variable—chronic disease (chr)—had a mean of 0.10. This indicates that approximately 10% of workers in the final sample self-reported being diagnosed by a physician with at least one chronic disease within the past 6 months. This proportion underscores chronic disease as a significant public health issue that cannot be overlooked. Regarding demographic characteristics, the sample’s average age was 38.42 years (SD = 10.38), indicating a predominantly young-to-middle-aged population. Gender distribution showed 58% males and 42% females. The mean marital status score of 1.86 corresponded to the “married” category in the classification variable, suggesting most individuals in the sample were in marital relationships. The average years of education (edu) was 10.74 years, roughly equivalent to a high school diploma, reflecting that the proportion of individuals with higher education qualifications in China may still be relatively limited. Regarding work characteristics, the average weekly working hours reached 52.47 h, far exceeding the national statutory 40-h standard workweek, clearly revealing the widespread phenomenon of “overwork” among the sample. This aligns with the work culture prevalent in certain industries during China’s current rapid economic development phase. Concurrently, only 62% of workers have signed labor contracts (LC mean = 0.62), indicating that a proportion of the labor market remains informal. The instability and lack of safeguards in such employment may pose threats to mental health. Regarding health behaviors, 32% of respondents reported smoking within the past month (smok), while 14% consumed alcohol more than three times weekly (drink). These behaviors themselves may serve as negative coping strategies for psychological stress, forming a complex bidirectional relationship with depressive symptoms. The mean subjective assessment of local income level (sincome) was 2.83, indicating a moderately low level. Meanwhile, the mean scores for income satisfaction (inc) and job safety satisfaction (saf) were 3.49 and 3.90, respectively, falling between “average” and “relatively satisfied.” This indicates that workers’ subjective assessments of their economic circumstances and job stability are generally neutral to positive. Crucially, social cognitive capital (ren), or generalized trust, showed that 58% of the sample tended to trust others. This proportion serves as a key indicator of social capital levels, with higher trust typically associated with better social integration and psychological well-being.

**Figure 2:**
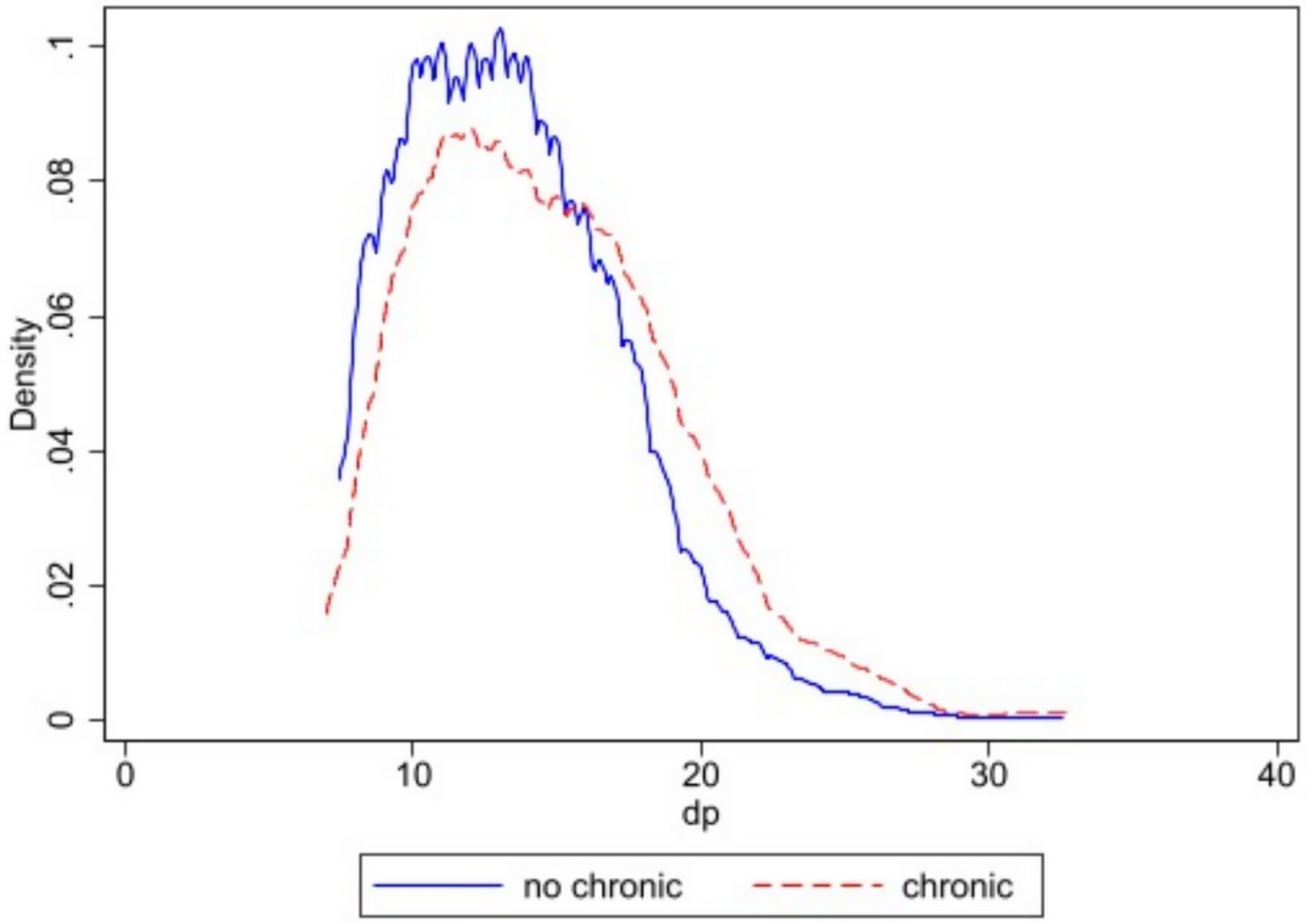
Kernel density estimation.

Figure 2 uses kernel density estimation (KDE) curves to compare the distribution of depressive symptoms scores between participants, employing a nonparametric approach. The figure clearly shows a significant distributional shift between the two density curves. The depression score density curve for the non-chronic disease group is overall shifted to the left of the coordinate axes, i.e., toward the lower score range. Its peak is higher and the curve steeper, indicating that depression scores within this group are more concentrated. Most individuals in this group exhibit relatively good mental health, with depression scores clustered in the lower range. In contrast, the density curve for the chronic disease group is markedly shifted to the right, with distribution skewed toward higher score regions. This curve exhibits a lower peak and a flatter, rightward-extending tail, revealing two key characteristics: First, depression scores within this group are less concentrated, indicating greater individual variation; Second, a substantial proportion of individuals fall into higher score ranges, indicating a significantly higher proportion of individuals at risk of moderate to severe depressive symptoms within the chronic disease patient group. Furthermore, the long tail on the right side of the curve signifies the presence of a non-negligible “high-risk” tail group within this cohort. These individuals exhibit particularly severe depressive symptoms and represent a population requiring focused attention and intervention (Table 2).

**Table 2:** Descriptive statistics.

Variable	Mean	SD	Min	p50	Max	*N*
Depressive symptom	13.65	3.880	8	13	32	7,896
Chronic disease	0.100	0.300	0	0	1	7,896
Age	38.42	10.38	18	37	60	7,896
Gender	0.580	0.490	0	1	1	7,896
Marital status	1.860	0.650	1	2	5	7,896
Education	10.74	4	0	12	23	7,896
Labor contract	0.620	0.490	0	1	1	7,896
Working hours	52.47	17.65	0.100	50	168	7,896
Smoking	0.320	0.470	0	0	1	7,896
Alcohol consumption	0.140	0.350	0	0	1	7,896
Local income level	2.830	0.910	1	3	5	7,896
Income satisfaction	3.490	0.980	1	4	5	7,893
Job safety satisfaction	3.900	0.920	1	4	5	7,894
Social cognitive capital	0.580	0.490	0	1	1	7,874

### 4.2 Depressive symptoms and chronic diseases

Table 3 reveals a positive association between chronic diseases and depressive symptoms in the baseline regression analysis. In the model without control variables, patients with chronic illness were significantly more depressive than those without chronic illness (OR = 1.590, *p* < 0.01). After the introduction of a range of control variables, the positive effect of chronic illness on depressive symptoms remained significant, with the OR rising to 1.671 and significant at the 1% level, suggesting that patients with chronic illness had a risk of depression that was approximately 67.1% higher than non-patients.

**Table 3:** Benchmark regression results.

Variables	(1)	(2)
	Depressive symptoms	Depressive symptoms
Chronic disease	1.590***	1.671***
	(0.114)	(0.128)
Age		0.985***
		(0.003)
Gender		0.814***
		(0.047)
Marital status		1.161***
		(0.044)
Education		0.963***
		(0.007)
Labor contract		0.934
		(0.047)
Working hours		1.008***
		(0.001)
Smoking		1.026
		(0.057)
Alcohol consumption		1.016
		(0.066)
Local income level		0.751***
		(0.018)
Occupation type	No	Yes
Observations	7,896	7,896

Models report log odds ratios; *, **, and *** denote statistical significance at the 10, 5, and 1% levels, respectively; the brackets in Columns are robust standard errors.

Among the control variables, age exhibited a negative association with depression (OR = 0.985, *p* < 0.01), indicating a slight decline in depressive symptoms with age; for gender, males were less depressive compared to females (OR = 0.814, *p* < 0.01); and higher levels of education were associated with lower depressive symptoms (OR = 0.963, *p* < 0.01), indicating a psychoprotective effect of education. Local income ratings correlated negatively with depression (OR = 0.751, *p* < 0.01).

### 4.3 Robustness test

First, both the ordered Probit and benchmark ordered Logit models fall under Generalized Ordered Response Models (GORMs). Structurally isomorphic, they are suited for ordered categorical dependent variables. Their key difference lies in the assumption regarding the random error term’s distribution: Logit uses a logistic distribution, while Probit assumes a standard normal distribution. Although parameter estimates are generally incomparable due to differing distribution scales, the direction and statistical significance of variable effects should be consistent. Thus, applying the Ordered Probit model serves as a sensitivity analysis concerning distributional assumptions. As displayed in Column 1 of Table 4, the coefficient of chronic diseases remains significantly positive at the 1% level, which confirms the robustness of the findings under different distributional assumptions.

**Table 4:** Robustness test results.

Variables	(1)	(2)
	Depressive symptoms	Depressive symptoms
Chronic disease	1.343***	1.110***
	(0.056)	(0.164)
Age	0.992***	−0.029***
	(0.001)	(0.006)
Gender	0.892***	−0.384***
	(0.029)	(0.121)
Marital status	1.090***	0.329***
	(0.023)	(0.082)
Education	0.978***	−0.086***
	(0.004)	(0.016)
Labor contract	0.959	−0.194*
	(0.027)	(0.106)
Working hours	1.005***	0.018***
	(0.001)	(0.003)
Smoking	1.011	0.037
	(0.032)	(0.116)
Alcohol consumption	1.021	0.077
	(0.037)	(0.138)
Local income level	0.849***	−0.624***
	(0.012)	(0.053)
Occupation type	Yes	Yes
Observations	7,896	7,896

The Oprobit model reports the log odds ratio, while the OLS model reports the correlation coefficient.

Second, the depression score—ranging broadly and evenly from 8 to 32—can be treated as an approximately continuous variable (40). Although the OLS model ignores the ordinal nature of the dependent variable, potentially introducing measurement error, its estimation results still serve as an important reference benchmark. If the significance of core variables remains consistent across different model specifications, interference from the dependent variable’s measurement scale on the conclusions can be largely ruled out. As illustrated in Column 2 of Table 4, the coefficient for chronic diseases remained statistically significant at the 1% level, indicating a consistent positive association with depressive symptoms across different model specifications.

Furthermore, the estimated results for control variables in both models showed high consistency with the benchmark model. The direction and significance of coefficients for variables such as age, gender, education, and working hours remained largely unchanged, indicating that model specification had minimal impact on the results and that the estimates were robust.

### 4.4 Mechanism test results

#### 4.4.1 Income satisfaction

Column 1 of Table 5 indicates that chr exert a negative impact on inc (OR = 0.726, *p* < 0.01). This finding supports theoretical expectations that chronic diseases often impose substantial economic pressures on patients through increased healthcare expenditures, reduced labor force participation, decreased working hours, or impaired work efficiency, thereby diminishing their income satisfaction. Further analysis reveals that income satisfaction itself exerts a negative influence on depressive symptoms (OR = 0.743, *p* < 0.01). Specifically, a one-unit increase in income satisfaction reduces the odds ratio for elevated depressive symptoms by approximately 25.7%.

**Table 5:** Mechanism test results.

Variables	(1)	(2)	(3)	(4)	(5)	(6)	(7)	(8)	(9)
	Income satisfaction	Depressive symptoms	Depressive symptoms	Job safety satisfaction	Depressive symptoms	Depressive symptoms	Social cognitive capital	Depressive symptoms	Depressive symptoms
Chronic disease	0.726***		1.601***	0.802***		1.634***	0.725***		1.604***
	(0.057)		(0.122)	(0.064)		(0.125)	(0.060)		(0.123)
Income satisfaction		0.743***	0.748***						
		(0.017)	(0.018)						
Job safety satisfaction					0.759***	0.762***			
					(0.020)	(0.020)			
Social cognitive capital								0.510***	0.516***
								(0.022)	(0.022)
Age	1.003	0.987***	0.985***	1.004	0.988***	0.985***	1.002	0.987***	0.985***
	(0.003)	(0.003)	(0.003)	(0.003)	(0.003)	(0.003)	(0.003)	(0.003)	(0.003)
Gender	1.215***	0.835***	0.834***	0.895*	0.799***	0.799***	0.937	0.791***	0.791***
	(0.080)	(0.048)	(0.048)	(0.059)	(0.046)	(0.046)	(0.065)	(0.046)	(0.046)
Marital status	1.007	1.165***	1.164***	1.010	1.164***	1.163***	0.964	1.160***	1.158***
	(0.040)	(0.044)	(0.044)	(0.042)	(0.044)	(0.044)	(0.041)	(0.044)	(0.044)
Education	1.003	0.965***	0.964***	0.988	0.963***	0.962***	1.071***	0.974***	0.972***
	(0.008)	(0.007)	(0.007)	(0.008)	(0.007)	(0.007)	(0.009)	(0.007)	(0.007)
Labor contract	1.025	0.937	0.938	1.137**	0.950	0.950	1.111*	0.954	0.952
	(0.055)	(0.047)	(0.047)	(0.062)	(0.047)	(0.048)	(0.063)	(0.048)	(0.048)
Working hours	0.994***	1.007***	1.007***	0.988***	1.007***	1.007***	0.996***	1.008***	1.008***
	(0.002)	(0.001)	(0.001)	(0.002)	(0.001)	(0.001)	(0.001)	(0.001)	(0.001)
Smoking	0.947	1.014	1.015	0.945	1.023	1.024	0.957	1.017	1.020
	(0.057)	(0.056)	(0.056)	(0.058)	(0.057)	(0.057)	(0.060)	(0.057)	(0.057)
Alcohol consumption	0.986	1.010	1.023	0.882*	0.987	1.000	0.931	1.007	1.018
	(0.069)	(0.066)	(0.067)	(0.063)	(0.065)	(0.065)	(0.068)	(0.066)	(0.066)
Local income level	2.074***	0.819***	0.822***	1.498***	0.779***	0.783***	1.144***	0.756***	0.760***
	(0.065)	(0.021)	(0.021)	(0.042)	(0.019)	(0.019)	(0.031)	(0.018)	(0.018)
Occupation type	Yes	Yes	Yes	Yes	Yes	Yes	Yes	Yes	Yes
Observations	7,893	7,893	7,893	7,894	7,894	7,894	7,894	7,894	7,894

Models report log odds ratios; *, **, and *** denote statistical significance at the 10, 5, and 1% levels, respectively; the brackets in Columns are robust standard errors.

More importantly, after simultaneously incorporating chronic disease and income satisfaction variables, the odds ratio for chronic disease affecting depressive symptoms decreased from 1.671 to 1.601 while remaining statistically significant. This implies that chronic diseases directly adversely affect mental health while also indirectly exacerbating depression by diminishing patients’ satisfaction with their income status.

#### 4.4.2 Job safety satisfaction

The findings in Column 4 of Table 5 suggest that chronic diseases exert a significant negative impact on job safety satisfaction (OR = 0.802, *p* < 0.01), suggesting that chronic conditions markedly reduce individuals’ satisfaction with workplace safety conditions. Further analysis indicates that job safety satisfaction itself has an independent negative effect on depressive symptoms (OR = 0.759, *p* < 0.01).

After controlling for both chronic disease and job safety satisfaction variables, the direct effect coefficient of chr on dp decreased from 1.671 in the baseline model to 1.634 (*p* < 0.01). Chronic disease not only directly exacerbates depressive symptoms but also indirectly impacts mental health by reducing patients’ subjective evaluations of workplace physical safety.

#### 4.4.3 Cognitive social capital

Column 7 of Table 5 indicates that chronic illness exerts a significant negative impact on social cognitive capital (OR = 0.725, *p* < 0.01). The disease state markedly reduces individuals’ fundamental trust in others and society. This effect likely stems from the chronic pain, functional limitations, and stigma associated with chronic illness. These factors collectively reduce patients’ social participation, diminish the quality of interpersonal interactions, and may even reinforce defensive and distancing tendencies due to experiences of discrimination or misunderstanding, ultimately eroding their sense of universal trust.

Social cognitive capital was also negatively associated with depressive symptoms (OR = 0.510, *p* < 0.01). Specifically, for each unit increase in an individual’s interpersonal trust level, the odds ratio for experiencing higher-level depression decreased by approximately 49%. After controlling for both chronic disease and interpersonal trust variables, the OR of chronic disease on depressive symptoms significantly decreased from 1.671 to 1.604 (*p* < 0.01), indicating that social cognitive capital partially mediated the influence pathway from chronic disease to depression. This finding reveals that chronic disease not only directly harms mental health but also indirectly increases depression risk by eroding patients’ trust in others through a social cognitive process.

### 4.5 Heterogeneity analysis

#### 4.5.1 Gender

As shown in Columns 1 and 2 of Table 6, chronic diseases significantly increase depressive symptoms in both males and females, although the magnitude of the effect varies. Specifically, among females, the odds ratio for depression among chronic disease patients was 1.546 times higher than among non-patients (OR = 1.546, *p* < 0.01). Among men, this ratio was even higher, with an odds ratio of 1.715 times (OR = 1.715, *p* < 0.01). This indicates that while chronic diseases pose a significant threat to mental health for both genders, their negative impact on male depression is slightly greater than on female depression.

**Table 6:** Heterogeneity results.

Variables	(1)	(2)	(3)	(4)	(5)	(6)
	Gender		Social support		Internet usage	
	Female	Male	Weak	Strong	No	Yes
	Depressive symptoms	Depressive symptoms	Depressive symptoms	Depressive symptoms	Depressive symptoms	Depressive symptoms
Chronic disease	1.546***	1.715***	1.708***	1.672***	1.783***	1.523***
	(0.204)	(0.164)	(0.213)	(0.166)	(0.176)	(0.199)
Age	0.991*	0.983***	0.986***	0.985***	0.980***	0.996
	(0.005)	(0.003)	(0.005)	(0.003)	(0.003)	(0.005)
Gender			0.845	0.791***	0.866*	0.722***
			(0.088)	(0.057)	(0.071)	(0.064)
Marital status	1.209***	1.105*	1.172***	1.143***	1.231***	1.003
	(0.072)	(0.058)	(0.071)	(0.058)	(0.059)	(0.066)
Education	0.951***	0.972***	0.976**	0.956***	0.945***	1.006
	(0.011)	(0.010)	(0.012)	(0.009)	(0.009)	(0.016)
Labor contract	0.836**	1.017	0.854*	1.025	0.912	0.964
	(0.066)	(0.069)	(0.070)	(0.069)	(0.055)	(0.092)
Working hours	1.010***	1.008***	1.006**	1.010***	1.006***	1.015***
	(0.002)	(0.002)	(0.002)	(0.002)	(0.002)	(0.003)
Smoking	0.953	1.034	0.961	1.083	1.002	1.118
	(0.273)	(0.060)	(0.094)	(0.077)	(0.071)	(0.109)
Alcohol consumption	1.205	1.029	1.036	1.031	1.007	1.118
	(0.272)	(0.071)	(0.123)	(0.087)	(0.076)	(0.152)
Local income level	0.761***	0.733***	0.723***	0.817***	0.777***	0.653***
	(0.029)	(0.025)	(0.030)	(0.027)	(0.022)	(0.032)
Occupation type	Yes	Yes	Yes	Yes	Yes	Yes
Observations	3,333	4,563	2,887	5,009	4,813	3,083

Models report log odds ratios; *, **, and *** denote statistical significance at the 10, 5, and 1% levels, respectively; the brackets in Columns are robust standard errors.

#### 4.5.2 Social support

This study measured social support using the single-item indicator “quality of personal relationships.” This binary variable was created by coding respondents as 1 if their unprocessed scores exceeded 6 (signifying greater support) and 0 otherwise (denoting lesser support). As illustrated in columns 3 and 4 of Table 6, chronic disease exerted a statistically significant positive impact on depressive symptoms across both social support subgroups, albeit with marked differences in the magnitude of this effect. In particular, within the group of people with limited social support, the likelihood of depression in those with chronic conditions was 1.708 times greater than in non-patients (OR = 1.708, *p* < 0.01). In contrast, among those with stronger social support, this incidence ratio was 1.672 times higher (OR = 1.672, *p* < 0.01).

#### 4.5.3 Internet usage

Internet usage, as a vital channel for information access and social participation in modern society, is increasingly recognized for its role in chronic disease management and mental health maintenance. This research classifies participants into two groups according to their answers to the question “Have you ever used the internet?”: internet users (coded as 1) and non-users (coded as 0). Among non-internet users, the odds ratio (OR) for depression occurrence among chronic disease patients was 1.783 times higher than among non-patients (OR = 1.783, *p* < 0.01). Among internet users, this OR decreased to 1.523 times higher (OR = 1.523, *p* < 0.01). These findings indicate that while chronic diseases significantly increase depression risk across all populations, their psychological impact is more severe among non-internet users.

## 5 Discussion

Using data from the 2022 China Family Panel Studies (CFPS), this research examined the association between chronic illnesses and depressive symptoms, as well as the underlying psychosocial mechanisms. Findings indicate that chronic diseases significantly elevate individuals’ risk of depression, a result that remains robust after controlling for a series of confounding variables and adjusting model specifications. More importantly, this study reveals three mediating pathways through which chronic diseases influence depressive symptoms: income satisfaction, job safety satisfaction, and social cognitive capital. Significant heterogeneous effects were also identified across different genders, levels of social support, and internet usage groups. The following sections provide a comprehensive discussion of these key findings.

First, this study found that individuals with chronic illnesses exhibit significantly higher depression risk than those without chronic conditions, consistent with existing research findings (4, 10, 11). This study further proposes and validates three influence pathways from a psychosocial perspective: financial stress exacerbates depression by reducing income satisfaction (41); occupational insecurity, increases depression risk by diminishing job safety satisfaction (42); and social isolation coupled with lack of trust indirectly causes emotional problems by damaging social cognitive capital (43).

Second, mechanism analysis indicated that income satisfaction, job safety satisfaction, and social trust partially mediated the association between chronic illnesses and depressive symptoms. Chronic disease patients, facing increased medical expenses and diminished work capacity, are more prone to dissatisfaction with their economic circumstances, which in turn triggers anxiety and depression (44, 45). Simultaneously, the threat chronic illness poses to employment stability significantly reduces patients’ job safety. This insecurity, is particularly amplified in China’s labor market, where long working hours and informal employment are prevalent (average weekly work hours: 52.47; only 62% of workers have formal contracts). Furthermore, chronic illness often leads to reduced social participation, “sickness stigma,” and diminished interpersonal trust, further diminishing social cognitive capital and thereby heightening depression risk (46). These three pathways collectively form a psychosocial transmission network, revealing the complex mechanisms through which chronic illness impacts mental health.

Heterogeneity analysis indicates that male chronic disease patients face a higher risk of depression than females (47), potentially linked to traditional cultural expectations of men as “breadwinners” and their emotional expression patterns. Among individuals with weaker social support networks, chronic diseases exert a more pronounced impact on depression, underscoring social support as a crucial buffer against the psychological toll of illness (48). As a vital psychosocial resource, social support offers multifaceted protection when individuals face health-related stress: First, at the emotional support level, care, understanding, and outlets for emotional expression from friends and family help alleviate negative emotions stemming from illness (49); Second, at the instrumental support level, practical assistance (such as accompanying patients to medical appointments or providing financial aid) alleviates tangible difficulties (50). Finally, at the informational support level, access to disease management knowledge and medical advice enhances patients’ sense of control (51). For those with weaker social support networks, the absence of these protective factors forces them to cope alone with the physical, mental, and financial burdens of chronic illness, leading to heightened depression risk (52). Notably, internet usage demonstrated a significant protective effect: chronic disease patients who used the internet exhibited markedly lower depression risk than non-users. The internet provides patients with channels for information access, social connection, and psychological adjustment, partially compensating for deficiencies in offline social support (53). Internet use may mitigate chronic diseases’ negative effects on mental health through multiple mechanisms. To begin with, it offers easy access to information, allowing patients to gain a better grasp of disease-related knowledge, treatment approaches, and self-management techniques—thereby boosting their sense of control and self-efficacy (54). Second, online platforms—such as social media, health forums, and support groups—offer spaces for social support and emotional exchange, helping alleviate loneliness and stigma associated with illness while promoting psychological adaptation (55). Additionally, the internet provides abundant recreational resources and online services (e.g., telemedicine, mental health courses) that help patients divert attention, relieve stress, and improve emotional well-being (56).

Several limitations are associated with this study. First, the depressive symptoms are self-reported, potentially introducing common method bias. Second, the data failed to identify the specific purpose of Internet use, making it difficult to completely rule out its negative effect of exacerbating the risk of depression, such as amplifying fear of illness through online information. Future research is necessary to differentiate between different types of Internet use in order to more fully assess their impact. Third, the findings of this paper need to be treated with more caution as the data used in this paper are cross-sectional and it is not possible to establish that there is a causal relationship between the variables.

Despite these limitations, the study offers both theoretical and practical value. The study integrates social psychology and health economics to propose a multiple mediation framework. Practically, this paper recommends greater attention to patients’ psychosocial needs in chronic disease management. This involves establishing a multidimensional mental health promotion system through enhanced economic security, strengthened employment support, increased social participation, and expanded healthy internet usage. Particularly in the digital age, governments and healthcare institutions can collaborate to develop online support platforms for chronic disease patients. These platforms ought to offer information, counseling, and community-based services to enhance psychological well-being and disrupt the illness-depression vicious cycle.

## 6 Conclusion

This study analyzes how chronic diseases affect depressive symptoms and underlying psychosocial mechanisms, using data from the 2022 China Family Panel Studies (CFPS). Findings indicate that chronic diseases significantly elevate workers’ risk of depression, with affected individuals exhibiting approximately 1.67 times higher incidence rates than non-affected counterparts. This result remains robust after controlling for multiple confounding variables and adjusting model specifications, confirming chronic diseases as a key predictor of depressive symptoms.

Further mechanistic analysis revealed that chronic diseases indirectly influence depressive symptoms through three psychosocial pathways: income satisfaction, job safety satisfaction, and social cognitive capital all partially mediate this relationship. Chronic diseases exacerbate depressive symptoms by intensifying economic pressures, weakening perceptions of job safety, and reducing interpersonal trust levels. Heterogeneity analysis reveals that the effect of chronic diseases on depression is stronger among males, individuals with low social support, and those lacking internet access. These findings highlight the importance of gender roles, social support networks, and digital resources in mitigating illness-related psychological distress.

This research broadens the understanding of chronic disease-depression comorbidity through a psychosocial lens. It underscores the importance of economic safety, improved work environments, rebuilding social trust, and internet-based health applications in chronic disease management. Future policy development should prioritize multidimensional psychosocial support, particularly in the digital context. By integrating online and offline resources, a more comprehensive mental health promotion system for chronic disease patients can be established. This approach will effectively break the vicious cycle of “disease-depression,” thereby enhancing patients’ quality of life.

## Data Availability

Publicly available datasets were analyzed in this study. This data can be found at: https://opendata.pku.edu.cn/dataverse/CFPS.

## Footnotes

^1^According to Article 15 of the *Labor Law of the People’s Republic of China*, there is a prohibition against employers employing adults under 16 years of age. This paper chooses a sample of 18 years of age or older because workers who are 16 years of age or older, but under the age of 18, are considered to be ‘underage workers’ and enjoy special protection. In addition, China’s delayed retirement policy, which will take effect from 1 January 2025, will not affect the legal working age during the sample period.
